# Infant and Baby Feeding and the Development of the Maxillofacial Complex Based on Own Observations and the Literature

**DOI:** 10.34763/devperiodmed.20182203.255259

**Published:** 2018-10-04

**Authors:** Dorota Cudziło, Dorota Pałczyńska, Magdalena Bednarczyk

**Affiliations:** 1 Maxillofacial Orthopedics and Orthodontics Department, Institute of Mother and Child, Warsaw Poland; 2 Neonatal Intensive Care Unit, Institute of Mother and Child, Warsaw Poland

**Keywords:** breastfeeding, bottle feeding, maxillofacial complex, stomatognathic development, motor patterns, karmienie piersią, karmienie butelką, kompleks szczękowo-twarzowy, rozwój układu stomatognatycznego, wzorce motoryczne

## Abstract

The method and technique of feeding a young child affect the shape of the maxillofacial complex. Breastfeeding is the recommended method of feeding in the first six months of life. It is encouraged to continue natural feeding in later months, simultaneously developing other food extraction techniques. The correct formation of the stomatognathic system is a result of the correct organization of the motor patterns during feeding.

## Streszczenie

Sposób i technika karmienia dziecka ma wpływ na kształtowanie się kompleksu szczękowo-twarzowego. Karmienie piersią jest rekomendowanym sposobem żywienia przez pierwszych sześć miesięcy życia. Zaleca się kontynuowanie karmienia naturalnego w kolejnych miesiącach życia, przy równoczesnym kształtowaniu się następnych technik pobierania pokarmu. Prawidłowe kształtowanie się układu stomatognatycznego jest efektem prawidłowej organizacji wzorców motorycznych, które są realizo-wane podczas karmienia.

## Background

A better understanding of the immuno-metabolic mechanism in children’s organisms induced by the bioactive components in the mother’s milk encourages the medical society, government and non-governmental organisations, including international ones, like WHO or UNICEF, to strongly endorse natural feeding. The need to naturally feed babies is commonly known, and its positive impact on physical and emotional development is well documented [1, 2, 3, 4, 5]. It is also believed, that natural feeding is beneficial for the growth and development of the stomatognathic system. The aim of this paper is to promote discussion regarding this topic on the basis of the PubMed literature review, as well as own experience.

### Infant feeding and stomatognathic physiology

Infant development is influuenced not only by the type of milk, but also by the means of its delivery. Extracting milk in the first months of life involves the act of suction, coordinated with swallowing and breathing. Although the kind of milk is the same in breastfeeding and breastmilk feeding, the mechanics is different.

During breastfeeding an important factor in extracting milk is creating underpressure inside the mouth. Geddes et al showed that when the infant’s tongue is lifted high towards the palate, the underpressure in their mouths is approximately -64 mmHg, and when the tongue is lowered, the underpressure rises to -145 mmHg and induces milk flow. During bottle feeding, milk extraction includes mainly compressing the teat. Bottle fed babies show lower oxygen saturation, create lower underpressure inside the mouth and are characterised by different patterns of suck-swallow-breathe coordination. Creating underpressure inside the baby’s mouth clearly impacts oxygen saturation, heart rate and suck-swallow-breathe coordination, but the impact of this is still unclear. [2]

Decidedly more oral muscles are involved in the breastfeeding process than in bottle feeding. Milk extraction phases are sequentially synchronised in both cases: they begin with opening the mouth, work of the lips, tongue, maxilla, palate and other orofacial structures, ending with swallowing and breathing. This function is accomplished periodically, with phase dissociation (functionally founded separation) of individual body parts involved in feeding. [6]

**Fig. 1:**
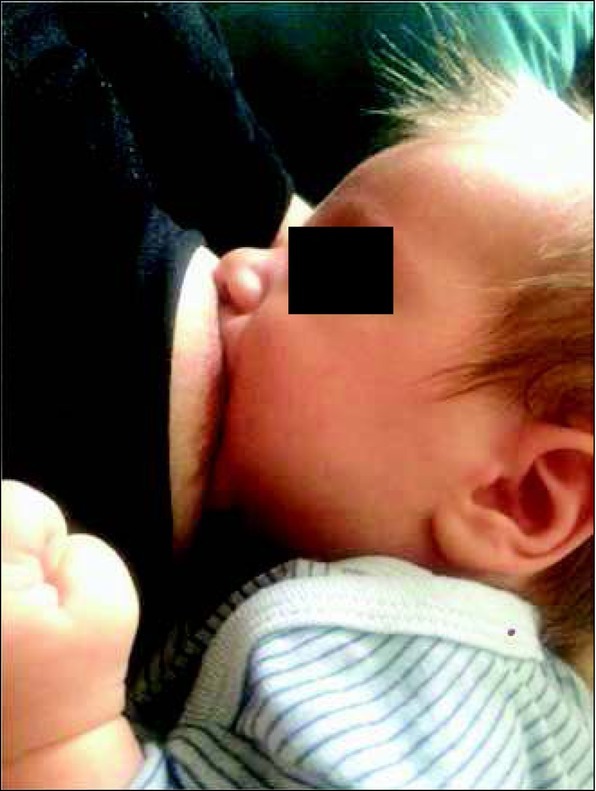
Correct position of the lips at the breast: wide open, tip of the nose and chin touching the breast. Own source. Ryc. 1. Prawidłowa pozycja warg przy piersi: szeroko otwarte, czubek nosa i brody oparty na piersi. Źródło własne.

**Fig. 2:**
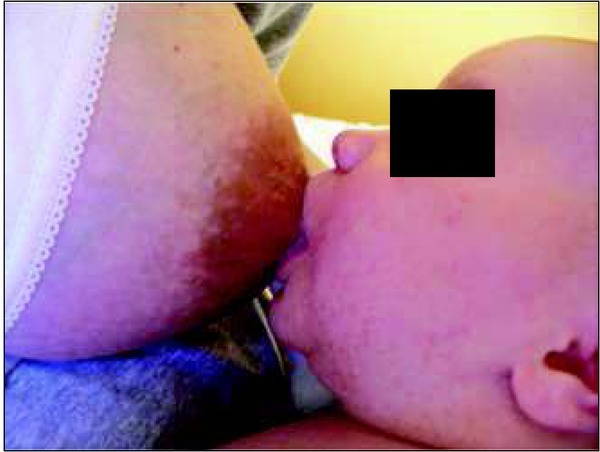
Incorrect position of the lips at the breast: narrow angle, shallow hold. Own source. Ryc. 2. Nieprawidłowa pozycja warg przy piersi: wąski kąt, płytki chwyt. Źródło własne.

The rhythm of suction, swallowing and breathing in both ways of feeding is modified relative to other factors, such as the baby’s age, hunger intensity, position of the mouth at the breast, the baby’s tiredness, and milk flow (Figure 1, Figure 2).

**Fig. 3:**
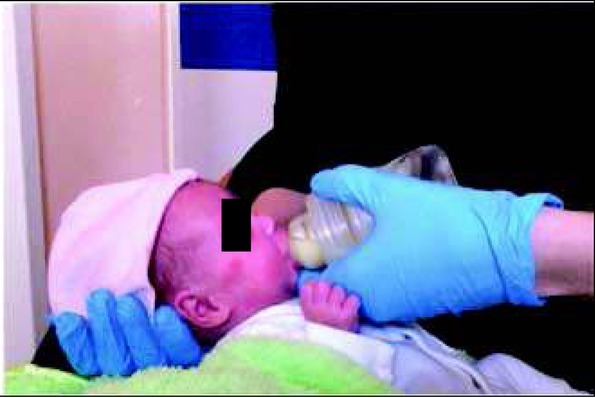
Correct position of the lips during bottle feeding: resting at the edge of the teat. Own source. Ryc. 3. Prawidłowa pozycja warg podczas karmienia butelką: oparte o kołnierz smoczka. Źródło własne.

**Fig. 4:**
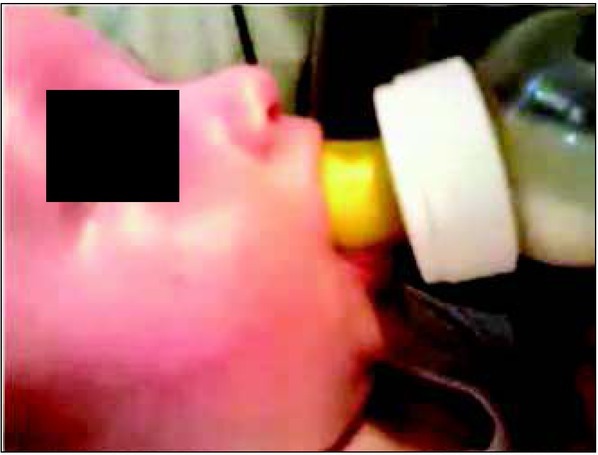
Incorrect position of the lips during bottle feeding. Own source. Ryc. 4. Nieprawidłowa pozycja warg podczas karmienia butelką. Źródło własne.

During bottle feeding, choosing the right teat and position are crucial (Figure 3, Figure 4).

**Fig. 5:**
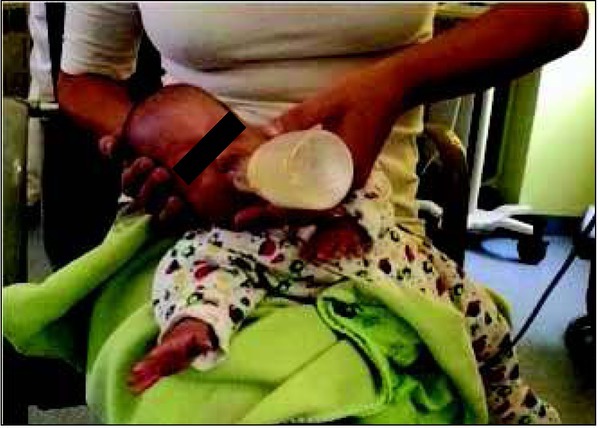
Lateral feeding position - recommended for premature newborns. Own source. Ryc. 5. Karmienie w pozycji bocznej: zalecane dla wcześniaków. Źródło własne.

Winnicka [7] points out specific teat parameters, such as the quality of material (silicon or rubber), size and shape of the hole, shape and dimensions of individual teat elements, hardness (if not of individual elements, then at least of the teat as a whole), the way of venting. The author verifies the views on bottle feeding and focuses on the quality of movement (correct protrusion of lips in order to seal the grip, symmetrical activity, flat and wide position of the tongue, underpressure caused by tongue work and mouth stabilisation), not on the feeding effectiveness viewed as the rate of milk flow. According to the author, neglecting the quality of movement results from the assumption that alternative ways of feeding contribute to speech defects and malocclusion. (Figure 5).

Moral et al published their study in 2011, comparing infants’ technique of suction depending on whether they were exclusively breast fed, exclusively bottle fed or combining both. They observed that exclusively bottle fed babies executed fewer suction moves than breastfed ones, with longer pauses. Suction mechanics in breast fed and bottle fed babies were individually patterned – the same number of suction moves during breast and bottle feeding, but fewer and shorter pauses [3].

Other researchers interested in the infant feeding method and stomatognathic physiology emphasize that during breast suction infants breathe through their noses, which strengthens the physiological breathing route between feedings. Simultaneously, the infant’s jaw is in a forced protraction and the gums are close, like in a bite.

This movement causes the whole stomatognathic muscle system to be involved in milk extraction [4].

During breastfeeding other muscles of the orofacial complex also work intensively – the cheeks, the lips and the tongue. When observing the act of suction, one must pay close attention to the frenulum, to rule out ankyloglossia. If a short frenulum restricts tongue mobility, it is recommended to cut it surgically in order to raise the effectiveness of breastfeeding [8, 9, 10].

Pires et al observed in their research that muscle stimulation through breastfeeding is beneficial for the masticatory system on later developmental levels. The authors examined children aged 3-5 regarding the masseter function and the length of natural feeding. It was shown that children breast fed for at least 12 months scored significantly higher in the quality of chewing, regardless of whether they were also bottle fed or sucked on pacifiers in early childhood [5].

Bottle fed babies present other mechanism of tongue work – its position depends on the material and hole size.

It should be noted, though, that the tongue’s position is lower than during breastfeeding. Moreover, the lips are wider apart. As a result of weaker stimulation of the tongue and mandible, masseter and pterygoid work is reduced, as the mere lowering of the tongue produces sufficient milk flow [4]. Some studies show, though, that masseter work in infants fed with a special teat bottle may be close to that at the breast. It was observed by Sakashita et al [11] in 1996, in their qualitative and quantitative study of myoelectrical activity in bottle fed and breastfed infants, with a chewing type bottle teat. The same authors showed in their earlier study (1995) that masseter work in infants fed with a regular sucking type bottle teat is reduced as compared to breastfed infants [12]. Many authors point to the fact that during bottle feeding there is no synchronization between swallowing and nose breathing, which may contribute to establishing an oral breathing route [5]

Infant feeding technique is significantly affecting the development of the maxillofacial structures. The growth and development of the infant’s craniofacial region is determined by genetic factors and physiological stimulation during breathing, sucking, swallowing and chewing [12]. The correct development of the maxilla and mandible is also impacted by muscular stimulation, as during muscle contraction the bone is pulled or pushed, which then leads to its proper growth [13].

Breastfeeding involves both the masseter, as well as the temporal and pterygoid muscles, which contributes to their developing correctly in terms of size and strength. Additionally, it stimulates the temporo-mandibular joint and frontal growth of the mandible [4]. Proper stimulation of masseters and the maxillonasal complex, beginning at the mother’s breast, is important for the future correct chewing function, which together with genetic and environmental factors directly influences maxillary and mandibular growth, shorting and stomatognathic muscles [5].

Cranial bone analyses from the past, when breastfeeding was the only way to feed a baby, show a minimum percentage of what could be recognized as malocclusion [13]. Larson (1983) examined medieval craniums of Swedish children with deciduous and mixed teeth, and Palmer (1998) studies prehistoric craniums from India and Native Americans. Both authors showed a low frequency of malocclusion and other disorders connected to poor sucking. In Palmer’s study, involving 600 skulls, 98% showed no occlusion defects [13, 14].

### Feeding method influence on stomatognathic pathophysiology

Chen et al showed that in infants that were not breastfed, or were breastfed for no longer than 6 months, the likelihood of developing the habit of sucking on a pacifier was quadrifold compared to babies breastfed for more than 6 months. The observations of Degan and Puppin-Rontani were similar. They found a linear relationship with breastfeeding and pacifier use: the shorter the breastfeeding, the longer the pacifier use.

There are also studies proving that early discontinuation of breastfeeding may result in insufficient craniofacial muscle activity, which negatively aftects swallowing, breathing, speech and may contribute to malocclusion [16].

**Fig. 6:**
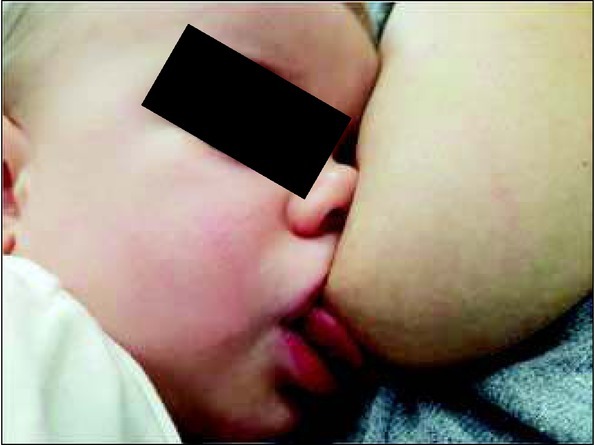
Incorrect tongue position during breastfeeding. Own source. Ryc. 6. Nieprawidłowa pozycja języka podczas karmienia piersią. Źródło własne.

It is worth pointing out that only the correct grasp of the breast and correct breastfeeding can ensure correct craniofacial development (Figure 6).

In the literature, it is signalled that some pathophysiological phenomena can be linked to the feeding method in infancy. One of these conditions is obstructive sleep apnea, OSA. Assuming that breastfeeding is essential to the proper swallowing mechanism, teeth alignment and palate structure, it also has to have an effect on airflow through the airways. Simultaneously, it is known that malocclusion is common in OSA patients [17].

### Malocclusion and bottle feeding

Studies show that in children who were breastfed for less than 6 months or never lateral cross bite and shortage of maxillary space are more common at the stage of deciduous teeth [18]. However, when the study group includes older children, at the early permanent teeth stage, researchers disagree. Abreu et al in 2016 published a systematic literature review. Out of 817 papers published until February 2015 and cited in the 8 most popular medical search engines, like Medline or PubMed, they chose 202 articles. Among them only 6 met the credibility criteria and examined the actual relationship between the method of infant feeding and the occurrence of malocclusion. The review outcome does not directly support any of the known hypotheses. One of the studies showed that in children breastfed for 6 months or longer there is greater protrusion of the lower incisors. Another study concerning prolonged breastfeeding linked to the more frequent occurrence of class II and III permanent teeth defects, three other papers showed no link between the method of feeding and the type of occlusion in mixed and permanent teeth. Only one of the six chosen papers proved that prolonged breastfeeding is linked to a lower risk of malocclusion at the mixed and permanent teeth stages [19]. A similar literature review was conducted on the relationship between the infant feeding method and the risk of malocclusion at the deciduous teeth stage [20]. In this paper Hermont et al observed some discrepancies in the relationship assessment between the feeding method and the occurrence of malocclusion, except for increased overjet mentioned in one of the articles cited.

Later papers, published after 1991, point to a more frequent occurrence in lateral crossbite in exclusively bottle fed children [12]. However, in spite of almost 1000 papers being reviewed and 223 chosen for analysis, only 10 of those, the cohort studies, were considered, due to the credibility inclusion criteria. As a result, Hermont et al decided that there is no evidence to support the link between bottle feeding and a greater risk of malocclusion in deciduous teeth. Still, they concluded: “It seems that breastfeeding might protect against malocclusion or promote correct occlusion development” [20].

## Conclusion

The infant and baby feeding method may affect the development of the maxillofacial complex. Various sucking mechanisms, differently engaging the stomatognathic elements in breastfed and bottle fed babies, as well as the teat type or feeding position, influence the system’s anatomic structure. Both in the case of breastfeeding and bottle feeding, after the first year of life, the child should have full control of spoon feeding, open cup drinking and solid food chewing, which positively affects craniofacial development.
